# Assessment of Hydroxyethyl Starch (6% HES 130/0.4) Kidney Storage in Critically Ill Dogs: A Post-mortem Prospective Study

**DOI:** 10.3389/fvets.2021.802507

**Published:** 2022-01-06

**Authors:** Katja-Nicole Adamik, Michael H. Stoffel, Simone Tangermann, Bettina de Breuyn Dietler, Nadine Stokar-Regenscheit

**Affiliations:** ^1^Division of Small Animal Emergency and Critical Care, Department of Veterinary Clinical Medicine, University of Bern, Bern, Switzerland; ^2^Division of Veterinary Anatomy, Department of Clinical Research and Veterinary Public Health, University of Bern, Bern, Switzerland; ^3^Department of Infectious Diseases and Pathobiology, Institute of Animal Pathology of the Vetsuisse Faculty, University of Bern, Bern, Switzerland; ^4^Division of Topographic and Clinical Anatomy, Institute of Anatomy, University of Bern, Bern, Switzerland

**Keywords:** synthetic colloids, AKI, osmotic nephrosis, tetrastarch, fluid therapy

## Abstract

**Objective:** Intravenous hydroxyethyl starch (HES) solutions are potentially nephrotoxic due to rapid renal tissue uptake, subsequent osmotic nephrosis, and long-lasting intracellular storage. This study aimed to investigate the severity of intracellular storage of HES in renal tissue samples from critically ill dogs receiving 6% HES 130/0.4.

**Materials and Methods:** Fresh, post-mortem (<2 h after death) renal tissue samples were analyzed through histology, immunohistochemistry (HES 130/0.4-specific antibodies), and electron microscopy for the severity of renal tubular vacuolization (VAC), intravacuolar HES accumulation (ACC), and ultra-structure impairment. Moreover, we investigated the relationship between VAC or ACC grade and HES dose (mL/kg), duration of HES administration (h), and pre-HES plasma creatinine concentrations.

**Results:** Histology revealed that 2/20 dogs (10%) had no, 11/20 dogs (55%) had mild, 5/20 dogs (25%) had moderate, and 2/20 dogs (10%) had severe VAC. Immunohistochemistry revealed that 5/20 dogs (25%) had no, 6/20 dogs (30%) had mild, 7/20 dogs (35%) had moderate, and 2/20 dogs (10%) had severe ACC. Both changes were predominantly found in the distal tubular epithelium of mild and moderate cases, and all tubular segments were affected in severe cases. Seven of 20 dogs (35%) had osmotic nephrosis (ON). On electron microscopy, large granules with an electron-dense content were repeatedly detected in individual cells, mainly in the distal tubules. No correlation was found between cumulative HES dose or duration of HES administration and VAC grade, ACC grade, or presence/absence of ON.

**Conclusion:** A high percentage of dogs had renal tubular HES storage and one-third of dogs showed HES-induced ON. Short-term HES administration caused VAC and ACC, regardless of the dose or duration of administration. In contrast to previous studies, HES 130/0.4 deposits were mainly located in the renal distal tubule.

## Introduction

Hydroxyethyl starch (HES) solutions belong to the class of synthetic colloids and comprise modified maize or potato starch suspended in an isotonic crystalloid solution (1). Since its introduction in the 1970's, several HES products with varying physicochemical properties, such as molecular weight (MW) and degree and pattern of hydroxyethylation, were developed. HES solutions were commonly used as an intravenous plasma expander to prevent or treat hypovolemia and hypotension in people and companion animals, including dogs, cats, and horses (2–5). However, coagulopathy, kidney injury, tissue storage, and pruritus are well-known side effects of HES in humans (6). To reduce these side effects, different HES preparations with improved physicochemical properties were developed, with the most recent low MW third-generation tetrastarch (HES 130/0.4 or 0.42) supposedly being the safest (Table 1). However, recent large-scale studies in people have found a higher incidence of acute kidney injury (AKI) and higher mortality even in patients receiving modern tetrastarch preparations (7–9).

**Table 1:** Common different HES types and their physicochemical characteristics.

**HES type**	**Classification**	**MW**	**MS**	**Generation**
Hetastarch	High	450–670	0.7 or 0.75	First
Pentastarch	Medium	200	0.5	Second
Tetrastarch	Low	130	0.4 or 0.42	Third

*MW, average mean molecular weight in kilo Dalton; and MS, molar substitution (number of hydroxyethyl residues attached to the glucose particles within the polymer)*.

After infusion, HES is cleared from the plasma both by renal excretion and by tissue uptake, and the tissue accumulation of HES molecules in humans and experimental animals is well-described. The most affected organs are the skin, kidney, liver, and bone marrow (10), with the highest HES concentrations detected in the kidney (11). Renal HES uptake occurs rapidly and induces osmotic nephrosis (ON), which is a mechanism underlying HES-induced AKI. Osmotic nephrosis refers to a histomorphological pattern with vacuolization and swelling of the renal tubular cells that compromise and occlude the tubular lumen with subsequent stasis of urine flow (12). Morphologic changes in ON can persist for years, but they are generally reversible, and functional recovery occurs after discontinuing treatment (12). Other mechanisms underlying HES-induced AKI include hyperviscosity-induced decreases in glomerular filtration rate, renal interstitial proliferation, macrophage infiltration, and tubular damage (13).

The evidence on HES-induced AKI in dogs is still contradictory. In three retrospective and one prospective study on critically ill dogs, either the absence (used product was 6% HES 130/0.4) (14–16) or presence (used product was 10% HES 200/0.5) (17) of AKI has been reported after administering HES. Two canine experimental studies (hemorrhagic shock model) found no difference in plasma or urinary AKI biomarkers (e.g., plasma and urinary neutrophil gelatinase-associated lipocalin, urine cystatin C, and kidney injury molecule-1) between dogs receiving 6% HES 130/0.4 and those receiving crystalloids (18, 19). These studies also found no significant differences in renal histopathology (e.g., proximal tubular microvesiculation) between dogs receiving HES, whole blood, or isotonic crystalloids (19). In contrast, in a postmortem histopathological examination of kidney tissue from dogs receiving 6% HES 670/0.75, the cumulative dose of HES was positively associated with renal tubular vacuolization severity.

Notably, the histomorphological pattern of ON is unspecific for HES. In addition to HES, other synthetic colloids (e.g., gelatin and dextran) and various other drugs are known to cause ON (e.g., intravenous immune globulins, mannitol, and radiocontrast preparations) (12). Therefore, it is essential to detect HES accumulation in patients with ON having a specific HES-antibody type. Tissue HES accumulation has been detected by light microscopy or electron microscopy. However, studies using immunohistochemical evaluation with specific HES antibodies are rare, and to the best of our knowledge, no such study on dogs has been performed.

This study aimed to evaluate fresh, post-mortem renal tissue samples from critically ill dogs that received 6% HES 130/0.4 by histology, immunohistochemistry using HES-specific antibodies, and electron microscopy. Moreover, the correlation between HES doses and duration of HES administration, along with the histomorphological pattern of ON, presence of HES in renal vacuoles, and plasma creatinine concentration, were investigated.

## Materials and Methods

### Ethics and Consent to Participate

The Veterinary Office of the Canton of Bern confirmed that according to the Swiss Federal Welfare Act of December 16th, 2005, no animal experimentation license was required. Signed owner consent was obtained before any post-mortem tissue sampling.

### Animals and Tissue Samples

Between April 2014 and April 2016, the dogs presented at the small animal clinic of the Vetsuisse Faculty that received 6% HES 130/0.4 (Volulyte, Fresenius Kabi AG, Oberdorf, Switzerland) during their hospital stay and subsequently died or were euthanized were eligible for inclusion. Only the tissue samples of dogs in which the time from death until final fixation of the organ sample was <120 min were included. Tissue samples were collected either by full necropsy, by target organ necropsy (kidney), or by true cut biopsies, depending on the owner's agreement.

### Clinical Data

After enrollment, an acute patient physiologic and laboratory evaluation (APPLE_fast_) score according to Hayes et al. (20) was retrospectively generated for each dog for the time of initiation of HES administration. The APPLE_fast_ score is a 5-variable, diagnosis-independent, illness severity score based on plasma concentrations of albumin and glucose, thrombocyte count, blood lactate level, and mentation score, with a maximum potential score of 50 (predicted probability of non-survival, 100%) (20). The total volume of HES (mL/kg) administered and duration of HES administration (bolus vs. constant rate infusion [CRI]) were recorded. Moreover, at the time of HES administration initiation, the heart rate (HR), systolic blood pressure, respiratory rate, rectal temperature, white blood cell counts (WBCs), percentage of band neutrophils, and plasma creatinine concentration were recorded. Accordingly, dogs were allocated to one or more of the following categories: shock (HR >160 bpm and shock index >1.0) (21); systemic inflammatory response syndrome (SIRS) (meeting at least two of the following criteria: hypo- or hyperthermia [rectal temperature <38.1 or >39.2°C], tachycardia [HR >120 bpm], tachypnea [respiratory rate >20 breaths per minute], increased WBCs (>16 × 10^9^ cells/L or leukopenia [ <6 × 10^9^ cells/L] and >3% band forms in the WBC count) (22); sepsis (SIRS plus known or suspected infection). Additionally, when available, plasma creatinine concentrations prior to HES and at the closest possible time point before death were recorded to compare pre- and post-HES creatinine concentrations.

### Post-mortem Histology and HES Immunohistochemistry

Fresh tissue samples were immediately fixed in 4% neutral buffered formalin, embedded in paraffin, sectioned at 4 μm, and stained with hematoxylin and eosin (HE). Immunohistochemistry (IHC) was performed on formalin-fixed and paraffin-embedded kidney tissue with a commercially available polyclonal rabbit antibody (Biorbyt, orb10857) raised against HES 130/0.40. Unspecific staining was blocked with 1% Bovine Serum Albumin in phosphate-buffered saline (Sigma-Aldrich, Merck, Darmstadt, Deutschland) for 10 min. The primary antibody was incubated at a 1:50 concentration at 4°C overnight. For detection, a DAKO® kit, comprising biotinylated goat anti-mouse and goat anti-rabbit IgG labeled streptavidin biotin (LSAB) reagents (DAKO *LSAB*®, DAKO, Glostrup, Denmark), was applied according to the manufacturer's instructions. Visualization was performed by an AEC substrate chromogen staining kit (AEC Staining Kit-Sigma-Aldrich, Merck, Darmstadt, Germany). Slides were counterstained with Ehrlich's hematoxylin and covered with coverslips in Aquatex® solution (Sigma-Aldrich, Merck, Darmstadt, Germany). Additionally, post-mortem pathological diagnosis was assessed in cases of full necropsies according to standard pathological procedures at the Institute of Animal Pathology, Vetsuisse Faculty, University of Bern, Switzerland. As a negative control, each sample was treated with the same procedure, except for the primary antibody. All samples were clean. As a positive control, a liver sample from a dog diagnosed with HES accumulation (storage disease) was added to every IHC batch run.

HE kidney sections were analyzed by two pathologists for histopathological lesions, focusing on morphological lesions of nephrotoxicity or ON. One section per patient was evaluated. Renal tubular vacuolization (VAC grade) severity was graded with a semi-quantitative scheme: 0, no vacuolization; 1, mild; 2, moderate; and 3, severe. IHC intracellular signal presence within the vacuoles in the renal tubuli as a measure for intracellular HES accumulation (ACC grade) was graded as follows: 0, none; 1, mild (focal positive signal in single tubular epithelial cells); 2, moderate (multifocal positive signal in tubular epithelial cells in up to 5 tubuli affected per high power field in 40x); and 3, severe (multifocal widespread positive signal in tubular epithelial cells; > 5 tubuli affected per high power field in 40x). Tubular ON was graded as either absent or present.

### Post-mortem Electron Microscopy

Electron microscopical analysis was performed on kidney samples from 11 dogs. Tissue samples were fixed in 2.5% glutaraldehyde in a 0.1 M cacodylate buffer (Sigma-Aldrich, Merck, Darmstadt, Germany) of pH 7.4 overnight and post-fixed with 1% OsO_4_ (Chemie Brunschwig, Basel, Switzerland) in a 0.1 M cacodylate buffer (Sigma-Aldrich, Merck, Darmstadt, Germany) for 2 h at room temperature, washed three times in cacodylate buffer, dehydrated in an ascending ethanol series and embedded in Epon (FLUKA, Buchs, Switzerland) resin, a mixture of Epoxy embedding medium, dodecenyl succinic anhydride (DDSA), and methyl nadic anhydride (MNA). Semi-thin sections were stained with toluidine blue and used to further narrow down regions of interest. Ultrathin sections of 70 nm were subsequently obtained with diamond knives (Diatome, Biel, Switzerland) on a Reichert-Jung Ultracut E (Leica, Heerbrugg, Switzerland) and collected on collodion-coated 200-mesh copper grids (Electron Microscopy Sciences, Hatfield, PA, USA). Sections were double-stained with 0.5% (w/v) uranyl acetate (Sigma Aldrich, Steinheim, Germany) for 30 min at 40°C and 3% lead citrate (Laurylab, Saint Fons, France) for 10 min at 20°C in an Ultrastain®° (Leica, Vienna, Austria) and examined in a Philips CM12 transmission electron microscope (Philips CM12 transmission electron microscope, FEI, Eindhoven, Holland) at an accelerating voltage of 80 kV. Micrographs were captured with a Mega View III camera using the iTEM software version 5.2 (Olympus Soft Imaging Solutions GmbH, Münster, Germany). Renal tubules were evaluated for luminal content as well as structural integrity and frequency of cell organelles. Post-mortem samples from two healthy dogs were used as controls.

### Statistical Methods

All statistical analyses were performed using a commercial software (MedCalc Software Ltd, B-8400 Ostend, Belgium). Variables were tested for normality using the D'Agostino-Pearson test for normal distribution. As data were not normally distributed, descriptive statistics were reported as median and range (min-max). An ordinal logistic regression model was performed with VAC grade as the dependent variable and cumulative HES dose (mL/kg), duration of HES administration (hours), and pre-HES plasma creatinine concentrations as explanatory variables. To determine the relationship of the ordinal variables VAC or ACC grade with HES dose (mL/kg), duration of HES administration (h), and pre-HES plasma creatinine concentrations, a Spearman's Rank Correlation Coefficient test was performed. For determining the relationship between the presence of sepsis and VAC or ACC-grade, a Chi-squared test was performed. To detect differences, specifically in VAC and ACC grades, between dogs receiving HES as a bolus or as a CRI, respectively, a Mann-Whitney test was performed. A *p* < 0.05 was considered statistically significant. An a priori sample size estimation was not performed.

## Results

### Cohort Characteristics

The kidney samples of 20 dogs were collected during the study period. Cohort characteristics and diagnosis are presented in Table 2. Dogs had a median age of 8.5 (range, 5–12) years and a median body weight of 32 (range, 10–46) kg. The median total APPLE_fast_ score was 32 (range, 20–39). Of the 20 dogs, 16 were humanely euthanized, and four died spontaneously. Cumulative HES dose, duration of HES administration, and HES application form (bolus vs. CRI) are summarized in Table 2. At the time of HES administration, 13/20 (62%) dogs were in shock, 19/20 (95%) had SIRS, and 8/20 (40%) had sepsis. Twelve dogs underwent full-body necropsy, and eight dogs underwent post-mortem renal biopsy sampling. The most frequent clinical diagnosis was a ruptured and metastatic hemangiosarcoma with hemoperitoneum leading to hypovolemic shock (*n* = 10; six were confirmed by necropsy and histopathology; the owners of four dogs declined necropsy). Overall, HES was administered as a bolus in 11 dogs, as a CRI in eight dogs, and as a combination of bolus and CRI in one dog. The median total bolus dose was 10 (range, 10–20) mL/kg, and the median total CRI dose was 79 (range, 6–270) mL/kg. The median duration of HES-CRI administration was 54 (range, 3–240) h.

**Table 2:** Patient characteristics, cause of death, diagnosis, total HES dose, HES application form, HES total application time, grading of renal tubular vacuolization (VAC grade), grading of renal HES accumulation (ACC grade), and the presence (+) or absence (–) of ON in 20 dogs.

**No**.	**Breed**	**Main diagnoses**	**Total dose**	**Application form**	**Application time (h)**	**VAC grade (by HE)**	**ACC-grade (by IHC)**	**ON**
1	Flat Coated Retriever, 10 y, mc	Splenic metastatic, ruptured HAS, HP^†^	10 mL/kg	Bolus	0.5	0	0	-
2	Cocker Spaniel, 12 y, mc	Spontaneous HP; metastatic neoplasia, and suspicion of HAS	15 mL/kg	Bolus	0.25	0	0	-
3	Flat Coated Retriever, 5 y, m	n/a	145 mL/kg	CRI	112	1	0	-
4	Border Collie, 9 y, fs	Bile peritonitis	30 mL/kg	CRI	72	1	0	-
5	Catalan Sheepdog 6 y, mc	Severe generalized colitis, pancreatitis, and pleural and abdominal effusion hypoalbuminemia	10 mL/kg	Bolus + CRI	2	1	0	-
6	French bulldog, 5 y, fs	Liver cirrhosis with necrosis; gastritis, acute, purulent, transmural; peritonitis, acute, and purulent^†^	120 mL/kg	CRI	60	1	1	-
7	German Shepheard, 8 y, mc	Gastric dilatation volvulus, post-surgical HP, and coagulopathy	6 mL/kg	CRI	6	1	1	-
8	American Staffordshire Terrier, 11 y, fs	Splenic metastasis, ruptured HAS, and HP^†^	10 mL/kg	Bolus	0.25	1	1	-
9	Golden Retriever, 8 y, m	Gastroenteritis, ulcerative, and chronic-active^†^	270 mL/kg	CRI	240	1	1	-
10	Golden Retriever, 12 y, mc	Spontaneous HP; metastatic neoplasia, and suspicion of HAS	10 mL/kg	Bolus	0.25	1	1	-
11	King Poodle, 8 y, mc	Colon rupture; peritonitis, fibrino-purulent, acute, severe, and diffuse^†^	20 mL/kg	Bolus	0.25	1	1	-
12	Border Collie, 5 y, fs	Hemorrhagic gastroenteritis, endotoxin shock; multifocal hemorrhages^†^	20 mL/kg	Bolus	0.25	1	2	-
13	Boxer, 10 y, fs	Splenic metastatic, ruptured HAS, and HP^†^	10 mL/kg	Bolus	0.25	1	2	-
14	Bullterrier, 8 y, fs	Necrotizing cholecystitis^†^	21 mL/kg	CRI	36	2	2	+
15	Mixed breed dog, 9 y, mc	Spontaneous HP, metastatic neoplasia, and suspicion of HAS	10 mL/kg	Bolus	0.25	2	2	+
16	Golden Retriever, 11 y, m	Splenic metastatic, ruptured HAS, HP; Gastric ulcers, acute^†^	10 mL/kg	Bolus	0.25	2	2	+
17	Labrador Retriever, 11 y, mc	Splenic, metastatic, ruptured HAS, and HP^†^	10 mL/kg	Bolus	0.25	2	2	+
18	German Shepheard, 6 y, m	Spontaneous HP; metastatic neoplasia, and suspicion of HAS	10 mL/kg	Bolus	3	2	2	+
19	Australian Shepheard, 12 y, fs	Splenic metastatic, ruptured HAS, and HP ^†^	10 mL/kg	Bolus	0.25	3	3	+
20	Pug, 6 y, fs	Wells syndrome and endotoxin shock^†^	50 mL/kg	CRI	48	3	3	+

† *Necropsy-based diagnosis; ON, osmotic nephrosis; HAS, hemangiosarcoma; HP, hemoperitoneum. The dogs are ordered in terms of renal alteration severity*.

### Plasma Creatinine Concentrations

The median plasma creatinine concentration prior to HES administration (available in 17 dogs) was 89 μmol/L (1.0 mg/dL) (range, 31–244 μmol/L; 0.35–2.77 mg/dL) (reference interval, 52–117 μmol/L; 0.6–1.3 mg/dL). Of these dogs, 12 had plasma creatinine concentrations within normal range (median, 73 μmol/L; range, 31–106 [median, 0.83 mg/dL; range, 0.35–1.20]), and five dogs had plasma creatinine concentrations above the normal range (median, 156 μmol/L; range, 120–244 [median, 1.77 mg/dL; range, 1.36–2.77]) prior to HES. Plasma creatinine concentrations at the time of death were available in five dogs (Table 3).

**Table 3:** Plasma creatinine concentrations pre- and post-HES, cumulative HES dose, duration of application, and histopathological results in 5/20 dogs.

**No**	**Total HES dose**	**Duration of application**	**Creatinine prior to HES**	**Creatinine time of death**	**Osmotic nephrosis**	**VAC grade (HE stain)**	**ACC grade (IHC stain)**
4	30 mL/kg	72 h	104 μmol/L (1.18 mg/dL)	49 μmol/L (0.56 mg/dL)	no	1	0
6	120 mL/kg	60 h	74 μmol/L (0.84 mg/dL)	173 μmol/L* (1.97 mg/dL)	no	1	1
9	270 mL/kg	240 h	79 μmol/L (0.90 mg/dL)	100 μmol/L (1.14 mg/dL)	no	1	1
14	21 mL/kg	36 h	64 μmol/L (0.23 mg/dL)	51 μmol/L (0.58 mg/dL)	yes	2	2
20	50 mL/kg	48 h	72 μmol/L (0.82 mg/dL)	237 μmol/L* (2.69 mg/dL)	yes	3	3

No. refers to the number of a specific dog as indicated in Table 1; the application form of HES in all five dogs was constant rate infusion; VAC grade, vacuolization grade assessed in hematoxylin eosin (HE)-stained slides, ACC grade, intravacuolar HES accumulation grade assessed in immunohistochemical (ICH)-stained slides; and *, creatinine concentrations above the RI.

### Renal Histopathology

**Figure 1:**
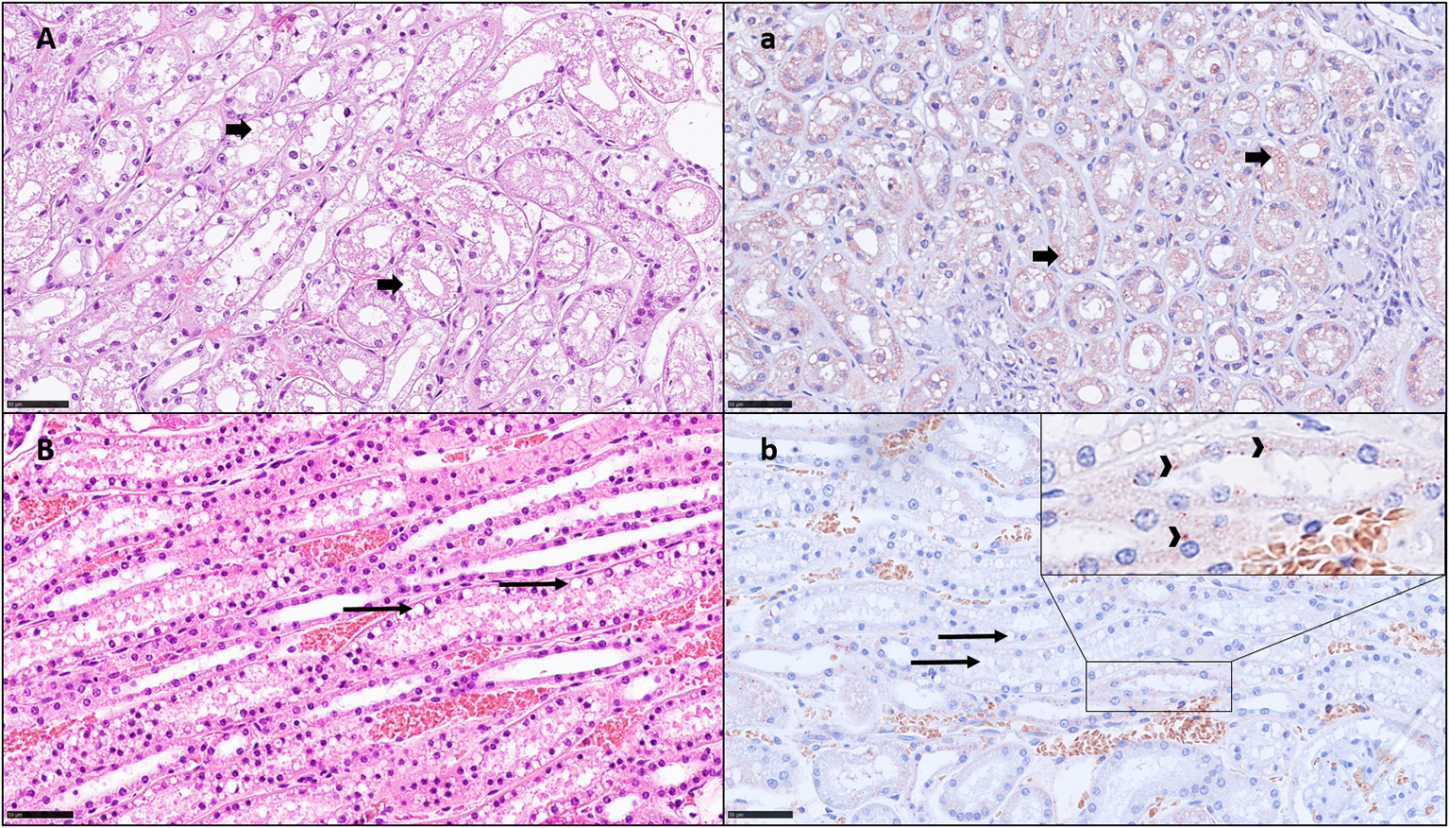
HE histology **(A,B)** and immunohistochemistry for HES **(a,b)** showing vacuolation in the kidney tubuli and positivity by HES IHC: **A**; **a**) Dog no. 20: Grade 3 VAC and ACC, small vacuoles (HE) with positive signal on IHC (short arrows). This kidney shows ON and HES IHC positivity in all segments. **B**; **b**) Dog no. 12: VAC and ACC Grade 1, with round clear vacuoles, mostly negative on IHC (long arrows) in the proximal tubuli but slightly positive in the distal tubuli (insert, arrow heads) bar = 50 μm.

Histopathological examination of the HE-stained slides revealed VAC grade 0 in 2/20 (10%) dogs, grade 1 in 11/20 (55%) dogs, grade 2 in 5/20 (25%) dogs, and grade 3 in 2/20 (10%) dogs (Table 2). In 16/20 (80%) kidneys, signs of vacuolization of the distal tubular epithelium were present, of which 7 (35%) were graded as 2 or 3 and were thus, interpreted as having ON (Table 2). In these dogs, ON was primarily present in the distal tubuli and less in the proximal tubuli (Figures 1A,B). No kidney showed lesions of ruptured epithelial cells or necrosis as a secondary event of ON. Additionally, signs of mild toxic or ischemic damage within the kidney (tubular degeneration and regeneration) were found in 3/20 (15%) dogs (number 2, 5, and 19). Three dogs had histological side lesions, though they were most likely clinically irrelevant. Dog number 12 had mild chronic pyelitis, dog number 9 had multifocal chronic infarcts, and dog number 4 had mild tubular mineralization. The other 17 dogs did not show any primary or secondary renal disease or similar histopathological lesions in the kidneys.

### Renal HES 130/0.4-Specific Immunohistochemistry

Histopathological examination of IHC-stained slides revealed ACC grade 0 in 5/20 dogs (25%), grade 1 in 6/20 dogs (30%), grade 2 in 7/20 dogs (35%), and grade 3 in 2/20 dogs (10%) (Table 2). Using IHC, a positive HES signal correlating with the vacuolization within the cytoplasm of these specific cells was observed during HE staining, predominantly in the distal tubular epithelial cells in mild and moderate cases (Figure 1B/b) and across all renal tubular segments in severe cases (Figure 1A). The IHC positive signal was present in numerous small vacuoles (Figure 1a), which were suspicious for small osmotic active vesicles. Larger, round, clear vacuoles in the proximal and distal tubuli seen by HE (Figure 1B) were in most cases negative for HES IHC (Figure 1b), indicating vacuolation of an origin other than HES. A summary of VAC and ACC scores in all 20 dogs is presented in Table 4.

**Table 4:** Summary of grading of distal tubuli epithelial vacuolization and HES accumulation in 20 dogs.

**Number of dogs**	**Interpretation**	**VAC grade (HE)**	**ACC grade (IHC)**	**Percentage of dogs**
*n* = 2	No changes	0	0	10%
*n* = 3 *n* = 6 *n* = 2	Mild	1 1 1	0 1 2	55%
*n* = 5	Moderate	2	2	25%
*n* = 2	Severe	3	3	10%

*VAC grade, vacuolization grade assessed in hematoxylin eosin (HE)-stained slides; and ACC grade, HES accumulation grade assessed in immunohistochemical (IHC)-stained slides*.

A significant correlation between VAC and ACC grades was found (*P* < 0.0001). No correlation was found between the APPLE_fast_ score and VAC (*P* = 0.41) or ACC grade (*P* = 0.23). There were no differences in VAC (*P* = 0.60) or ACC (*P* = 0.61) scores between dogs that received HES as a bolus compared to those that received it as CRI. There was no correlation between cumulative HES dose and VAC grade (*P* = 0.93), ACC grade (*P* = 0.46), or presence/absence of ON (*P* = 0.46), between duration of HES 130/0.4 administration and VAC grade (*P* = 0.83), ACC grade (*P* = 0.19), or presence/absence of ON (*P* = 0.54), or between pre-HES creatinine concentration and VAC grade (*P* = 0.98), ACC grade (*P* = 0.77) or presence/absence of ON (*P* = 0.47). There was no relationship between the presence or absence of sepsis and VAC (*P* = 0.67) or ACC grade (*P* = 0.97). Based on the ordinal logistic regression model, the cumulative dose of HES, pre-HES plasma creatinine concentrations, and duration of HES administration were not significantly associated with VAC grade.

Plasma creatinine concentrations pre- and post-HES administration (available in five dogs), cumulative HES dose, HES application time, and histopathological results are presented in Table 3.

### Renal Transmission Electron Microscopy

**Figure 2:**
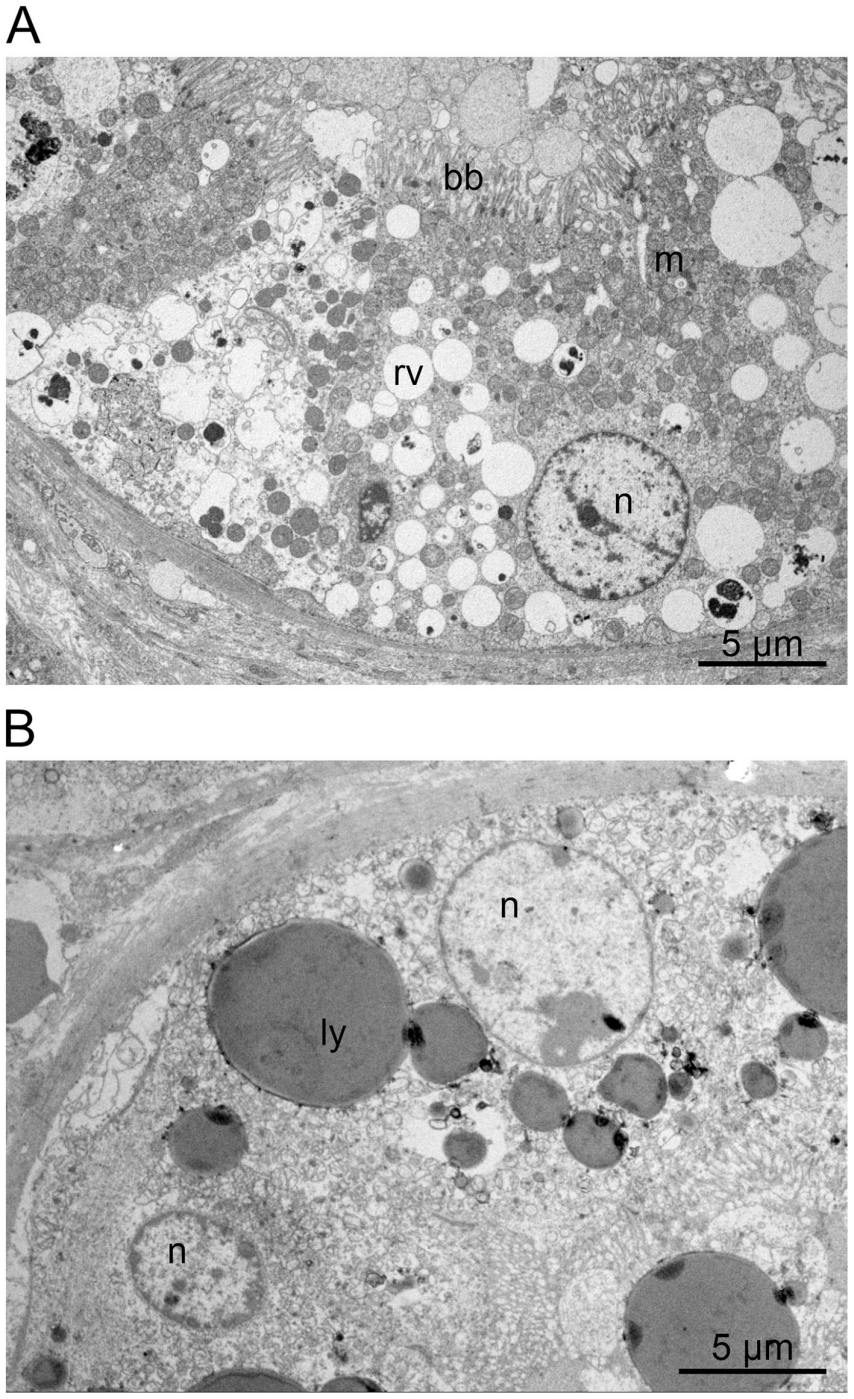
**(A,B)** HES histology. Renal transmission electron microscopy: Besides rounded, euchromatin-rich nuclei (n), the proximal tubule cells showed a typical brush border (bb) and numerous mitochondria (m). The cells also displayed an extensive endocytic-lysosomal apparatus with variable amounts of resorptive vacuoles (rv) and large spherical lysosomes (ly) containing homogeneous, electron-dense material. A similar endocytic apparatus was also noted in the distal tubules (not shown).

Ultrastructural detail preservation varied considerably within the tissue samples (Figure 2A). Overall, the rounded nuclei presented a regular, euchromatin-rich appearance with a peripheral rim and scattered dots of heterochromatin as well as clearly delineated nucleoli. In well-preserved areas, proximal tubules showed a typical brush border and basolateral interdigitations. Distal tubules also displayed a well-developed basal labyrinth and numerous mitochondria. In both proximal and distal tubules, an extensive endocytic-lysosomal apparatus with variable amounts of resorptive vacuoles was evident. However, the endocytic vesicles were never seen to completely fill out the cytoplasm or indent the nuclei. Large spherical granules with a homogeneous, electron-dense content were repeatedly detected in individual cells (Figure 2B). The presence and number of these granules was inconsistent and even varied between neighboring cells. The diameter of these granules often amounted to several micrometers. They were present in both proximal and distal tubules but more prominent in distal tubules and occasionally distorted the cell nucleus. Similar granules were also noticed in samples from two healthy dogs without HES therapy, albeit in smaller numbers. Moreover, some cells exhibited prominent membrane-bound organelles containing heterogeneous electron-dense material within a low-density matrix. Cellular debris was often seen in the lumen.

## Discussion

This is the first study prospectively evaluating the kidney samples of critically ill dogs receiving 6% HES 130/0.4 using histopathology, immunohistochemistry with HES 130/0.4-specific antibodies, and electron microscopy. Our major findings were that in 90% of the kidney samples, either low, moderate, or severe degree of vacuolization and/or intravacuolar HES accumulation was detected and that in only 10% of the samples, any HES-associated lesions were found. All dogs with ON showed a positive IHC signal associated with tubular vacuolation, indicating HES as a cause of ON. An uncommon finding is the location of HES-positive vacuoles, as they were predominantly found in the distal tubules in mild and moderate cases and across all tubular segments in severe cases. Usually, ON is described as vacuolization and swelling of the proximal tubules, whereas distal tubules and collecting ducts are usually unaffected (23, 24). Furthermore, HES (25–27) and other plasma expanders (e.g., dextran) or hypertonic solutions (e.g., mannitol, glucose, and sucrose) (12–28) are described to involve mainly the proximal tubular epithelial cells in people. Few data about renal tubular HES storage and its localization in dogs are available. Moreover, studies detecting HES molecules by specific antibodies in dogs are lacking. In a randomized experimental trial, 18 dogs received either a polymerized hemoglobin preparation or 6% HES 200/0.5 for complete blood exchange (dilution of hematocrit down to <5%). Only dogs in the HES group had renal proximal tubular epithelial cell vacuolization detected by light and electron microscopy (29). In the second study, dogs were subjected to hemorrhage for 60 min and were resuscitated with 20 mL/kg of fresh whole blood, 6% HES 130/0.4, 4% succinylated gelatin, or 80 mL/kg of isotonic crystalloid over 20 min. They were euthanized 3 h later. Renal histology revealed predominantly absent or mild tubular injury and microvesiculation in the proximal tubular epithelial cells after HES, as in the dogs that received isotonic crystalloid or whole blood (19). Notably, in dogs that received gelatin, microvesiculation was scored as “marked” in the proximal tubular epithelial cells. The third study is a retrospective study evaluating post-mortem kidney tissue samples from critically ill dogs that received 6% HES 650/0.75 and other hyperosmolar agents, such as dextran and mannitol. In that study, renal tubular vacuolization in proximal tubular cells, but not in the distal tubules, and collecting ducts was found (30). Two studies on kidney donors comprising people who received HES as a plasma-volume expander found ON in both the proximal and distal tubule (31, 32). It is unclear whether accumulation in the distal tubule has different effects on renal function than accumulation in the proximal tubule. Whether in our study the proximal epithelial tubular cells were able to degrade the HES 130/0.4 molecules and whether HES 130/0.4 was not absorbed by the proximal tubules, but the distal tubules, is unclear. The authors cannot conclusively determine the reason for the unusual localization in the mild and moderate cases.

To better assess cellular ultrastructure, samples were also evaluated by electron microscopy. This corroborated that many cells displayed a considerable number of vacuoles. However, in the studied samples, these vacuoles never filled the cytoplasm completely nor did they displace or distort the nuclei as described by Dickenmann et al. (12). Therefore, the findings were deemed as evidence of extensive endocytosis, occasionally resulting in ON as revealed by light microscopy and IHC. This aligns well with the occurrence of lysosomal granules. These observations support the contention of an extensive endocytic-lysosomal apparatus, which is likely to be associated with the uptake of HES. The cell debris seen in the tubular lumina, as well as the presence of phagosomes, may be indicative of some cellular decay. Strikingly, even immediately adjacent tubules or cells often revealed an extremely dissimilar ultrastructure. However, this observation has previously been reported by Dickenmann et al. (12). In summary, no qualitative but at most quantitative differences were observed at the ultrastructural level between samples from treated dogs and those from two healthy, untreated dogs.

Several underlying mechanisms for HES-induced AKI are described as follows: colloid-induced increases in plasma colloid-osmotic pressure and subsequent decreases in filtration pressure and glomerular filtration rate (“hyperoncotic AKI”); accumulation of HES-molecules in tubular epithelial cell lysosomes leading to renal epithelial dysfunction; tubular hyperviscosity and colloid precipitation; forming of occluding casts; and ON (1). These mechanisms can be reversible, and function may be restored (12); however, such factors may also be a first step in the development of irreversible cell lesions. A potential factor influencing the storage in renal tubular epithelial cells is the speed of HES degradation. Dogs possess a higher plasma α-amylase activity and faster HES degradation ability compared to humans (33), which may help reduce side effects before entering different immune and tissue cells. However, the intracellular accumulation of smaller HES molecules still occurs due to the absence of intracellular enzymes capable of metabolizing these substances (34). Whether and how HES degradation occurs inside the cells has not been fully elucidated yet. Most probably, the lysosomal enzyme acid α-glucosidase, which is necessary for the intralysosomal degradation of glycogen, degrades starch down to glucose (10, 23). The extent to which acid α-glucosidase can metabolize HES molecules is unknown, and no reports on acid α-glucosidase's ability to metabolize HES have been published. It cannot be excluded that the renal intracellular HES accumulations found in the present study could have been degraded over time in some dogs. However, the proven long-lasting accumulation of HES molecules in various cell types indicates that acid α-glucosidase is very inefficient, perhaps partly due to the chemical modification of HES to be resistant to degradation (10, 35).

Nine of the 20 included dogs received a dose of 10 mL/kg of HES once and were euthanized within 2 h after administration. The kidney changes were interpreted as mild in 3/9 dogs, as moderate in 4/9 dogs, and as severe in 1 dog. One dog had neither VAC nor ACC. Despite an identical HES dose and a similar period in which HES had time to distribute and accumulate in the body, kidney changes were quite diverse. This could be partly due to a different degree of perfusion impairment in these dogs, affecting the HES-uptake of tissue cells. This could also be due to impaired degradation as a consequence of cellular hypoxia. Nevertheless, our results confirm that the accumulation of HES 130/0.4 in canine renal tubular cells can occur rapidly (i.e., within 2 h). Similarly, intracellular vacuoles in hepatic Kupffer cells in people were found within 30 min after the intraoperative infusion of 1 g/kg of HES 450/0.7 by light and electron microscopy (36).

Eight of the 20 included dogs fulfilled the criteria for sepsis, which is a well-known risk factor for the development of organ failure and AKI, respectively. Among other factors, renal tubular epithelial dysfunction and increased glomerular permeability appear to be contributors to AKI (37). Consequently, larger HES molecules will be filtrated, resulting in more pronounced tubular resorption and vacuolar cell alterations (38). Accordingly, HES was found to significantly increase the risk for AKI in people with sepsis and septic shock (7, 8, 39). However, in a retrospective evaluation of the effects of HES 130/0.4 on plasma creatinine concentration in critically ill dogs, a subgroup analysis of septic dogs revealed that these dogs did not have a higher risk of HES-associated AKI than non-septic dogs (15). This result was also found in the present study. However, due to the small number of cases, results have to be interpreted with caution, and larger studies are needed to assess the risk of HES-induced AKI in dogs with sepsis.

In our study, there was no correlation between the cumulative dose or duration of administration and VAC or ACC grade. Our results contradict the results of the study by Schmid et al. (30), who found the cumulative HES dose (6% HES 670/0.75) to be a significant predictor of renal tubular vacuolization severity. However, no HES specific antibodies were used in their study, and it is difficult to exclude that other factors (e.g., post-mortem autolysis or hypoxic kidney injury) contributed to renal tubular vacuolization (30). Indeed, it has been advocated that HES solutions with lower MW and MS (such as HES 130/0.4) undergo more rapid urinary excretion and plasma clearance, indicating possibly reduced tissue uptake and storage. Our study results seem to confirm that. However, contrary to these postulated safety benefits, one study found that the average measured serum concentration of small HES molecules (<60 kilo Dalton) is 2.2 times higher over the first 24 h after infusion of HES 200/0.5 than over the first 24 h after infusion of HES 450/0.7 (40). Additionally, a meta-analysis found that lower MW and MS actually increased tissue uptake (41). Accordingly, a type II error may have been the reason for the lack of a relationship between cumulative HES dose or duration of administration and VAC or ACC grade. Therefore, our results should be attentively interpreted. Further investigations on the influence of MW and MS on HES uptake, storage, and processing in the kidney are warranted based on our data, the authors are unable to comment on the association between HES accumulation and the development of azotemia as the creatinine value prior to and after HES administration was available in only five dogs, and azotemia was present in only two dogs at the time of death. One of these two dogs had high VAC and ACC scores and ON. This dog suffered from acute eosinophilic dermatitis with edema [Wells-Like Syndrome (42)] and sepsis, received a total dose of 50 mL/kg over 2 days, and developed azotemia over 2 days. The authors cannot conclusively clarify whether there is a causal relationship between renal tubular vacuolar changes and azotemia.

This study has some limitations. Our study sample of only 20 dogs was very small, and a type II error is likely. Furthermore, the spectrum of underlying diseases, volume of HES, and duration of HES administration were diverse. As many of the dogs included were critically ill, it cannot be excluded that the degree of HES deposition was partly due to the underlying disease, although sepsis had no effect on VAC or ACC grade. It can be speculated that the intracellular degradation of HES is impaired due to cellular hypoxia as a consequence of critical illness and organ malfunction. As dogs received HES over a maximum of some days (some only as a bolus followed by euthanasia within a few hours), it remains unclear how much of the HES molecules would have been degraded if dogs would not have been euthanized or died. At 30–60 days after HES administration, no histological evidence of HES was found in experimental dogs and rats (43).

In conclusion, the present study shows that a high percentage of dogs had renal epithelial cell HES storage, and one-third showed HES-induced ON. The already short-term administration of HES caused renal tubular vacuolization and HES accumulation. Contrary to previous studies, HES 130/0.4 deposits were mainly located in the distal tubules in mild and moderate cases but were present in all tubular segments in severe cases. To evaluate chronic renal damage due to the accumulation of HES 130/0.4 molecules within the kidneys, further long-term studies are needed.

## Data Availability Statement

The original contributions presented in the study are included in the article/supplementary material, further inquiries can be directed to the corresponding author/s.

## Ethics Statement

The animal study was reviewed and approved by the Veterinary Office of the Canton of Bern. Written informed consent was obtained from the owners for the participation of their animals in this study.

## Author Contributions

K-NA, NS-R, and MS designed the study, collected and analyzed data, and wrote the manuscript. BB performed HE and IHC staining and provided technical support. NS-R and ST performed HE and IHC light microscopic evaluations. MS performed electron microscopic evaluations. All authors contributed to read and approved the final manuscript.

## Funding

This study was funded by Albert-Heim-Stiftung, Bern, Switzerland.

## Conflict of Interest

The authors declare that the research was conducted in the absence of any commercial or financial relationships that could be construed as a potential conflict of interest.

## Publisher's Note

All claims expressed in this article are solely those of the authors and do not necessarily represent those of their affiliated organizations, or those of the publisher, the editors and the reviewers. Any product that may be evaluated in this article, or claim that may be made by its manufacturer, is not guaranteed or endorsed by the publisher.

## Abbreviations

ACC: intracellular HES accumulation
AKI: acute kidney injury
APPLE: acute patient physiologic and laboratory evaluation
CRI: constant rate infusion
HES: hydroxyethyl starch
HR: heart rate
ICH: immunohistochemistry
ON: osmotic nephrosis
SIRS: systemic inflammatory response syndrome
VAC: renal tubular vacuolization
WBC: white blood cells.
